# Focused ultrasound enhanced intranasal delivery of brain derived neurotrophic factor produces neurorestorative effects in a Parkinson’s disease mouse model

**DOI:** 10.1038/s41598-019-55294-5

**Published:** 2019-12-18

**Authors:** Robin Ji, Morgan Smith, Yusuke Niimi, Maria E. Karakatsani, Maria F. Murillo, Vernice Jackson-Lewis, Serge Przedborski, Elisa E. Konofagou

**Affiliations:** 1 Department of Biomedical Engineering, Columbia University, New York, New York USA; 2 Department of Radiology, Columbia University, New York, New York USA; 3 Department of Pathology & Cell Biology, Columbia University, New York, New York USA; 4 Department of Neurology, Columbia University, New York, New York USA; 5 Department of the Center for Motor Neuron Biology and Disease, Columbia University, New York, New York USA; 6 Department of the Columbia Translational Neuroscience Initiative, Columbia University, New York, New York USA

**Keywords:** Drug delivery, Blood-brain barrier, Biomedical engineering, Neurotrophic factors, Parkinson's disease

## Abstract

Focused ultrasound-enhanced intranasal (IN + FUS) delivery is a noninvasive approach that utilizes the olfactory pathway to administer pharmacological agents directly to the brain, allowing for a more homogenous distribution in targeted locations compared to IN delivery alone. However, whether such a strategy has therapeutic values, especially in neurodegenerative disorders such as Parkinson’s disease (PD), remains to be established. Herein, we evaluated whether the expression of tyrosine hydroxylase (TH), the rate limiting enzyme in dopamine catalysis, could be enhanced by IN + FUS delivery of brain-derived neurotrophic factor (BDNF) in a toxin-based PD mouse model. Mice were put on the subacute dosing regimen of 1-methyl-4-phenyl-1,2,3,6-tetrahydropyridine (MPTP), producing bilateral degeneration of the nigrostriatal pathway consistent with early-stage PD. MPTP mice then received BDNF intranasally followed by multiple unilateral FUS-induced blood-brain barrier (BBB) openings in the left basal ganglia for three consecutive weeks. Subsequently, mice were survived for two months and were evaluated morphologically and behaviorally to determine the integrity of their nigrostriatal dopaminergic pathways. Mice receiving IN + FUS had significantly increased TH immunoreactivity in the treated hemisphere compared to the untreated hemisphere while mice receiving only FUS-induced BBB opening or no treatment at all did not show any differences. Additionally, behavioral changes were only observed in the IN + FUS treated mice, indicating improved motor control function in the treated hemisphere. These findings demonstrate the robustness of the method and potential of IN + FUS for the delivery of bioactive factors for treatment of neurodegenerative disorder.

## Introduction

Advances in treatment of neurodegenerative disease have been greatly hindered due to the crucial, but obstructive, blood-brain barrier (BBB), which prevents majority of therapeutic drugs from entering the brain parenchyma through systemic circulation^1^. Current methods of drug delivery to the brain, such as direct injection, convection-enhanced delivery and osmotic BBB disruption, are targeted but highly invasive, making them non-ideal for chronic administration^2^. Noninvasive techniques such as molecular modification of drugs and formulation of brain-penetrating nanoparticles offer a safer alternative, but do not provide a targeted effect within the brain^3,4^. One such noninvasive drug delivery technique that has gained attention is intranasal (IN) delivery^5^.

IN administration of drugs for systemic delivery has been used successfully in many clinical applications, such as influenza vaccine delivery^6^, but recently has generated interest for delivery to the central nervous system (CNS). IN delivery has been shown capable of allowing molecules direct and fast access to the brain, circumventing the BBB^7^. Once absorbed across the nasal epithelium, IN delivered drugs are transported along the olfactory and trigeminal nerves, which innervate the olfactory bulb and the brainstem, respectively^8^. Distribution of molecules to the rest of the CNS is transported along the cerebral perivascular space via convective transport such as bulk flow, through a phenomenon known as the “perivascular pump”^9,10^. IN delivery offers a simple, fast, and noninvasive drug delivery method that can be easily implemented and applied for chronic administration. Nevertheless, IN delivery has its own shortcomings including low delivery efficiency due to mucosal clearance and nasal absorption, and rapid widespread distribution throughout the brain, resulting in non-focal delivery^11,12^.

To overcome the shortcomings of IN delivery, some groups have focused on enhancing delivery efficiency across the nasal epithelium. The use of nanoparticles^13^, cell-penetrating peptides^14^, and gels^15^ has been investigated as formulations to carry biologics of interest for increased drug uptake and decreased mucosal clearance in the nasal passages. Others have investigated the use of different agents, such as matrix metalloproteinase-9 to increase the permeability and absorption across the nasal epithelium^16^. However, few have investigated ways to improve the non-focal delivery within the brain. Previous studies from our group have proposed a new method to enhance and focus IN delivery within the brain. By combining FUS-mediated BBB opening with IN delivery of molecules, we are able to provide targeted delivery of intranasally administered molecules within the brain. FUS-enhanced IN delivery (IN + FUS) has been shown to increase delivery efficiency of both fluorescently labeled dextran^17^ and brain-derived neurotrophic factor (BDNF)^18^ in targeted regions when compared to IN delivery alone, and was shown comparable in delivery efficiency to the traditional route of drugs delivered intravenously (IV) after FUS mediated BBB opening. During the FUS-mediated BBB opening, systemically circulating microbubbles (MBs) that are intravenously injected begin to cavitate when they reach the FUS beam path in the targeted region. The cavitating MBs can cause expansion and contraction of the surrounding microvasculature^19^, including the surrounding perivascular space, which may mimic the “perivascular pump” action, but at a much higher frequency and a more focal area. Furthermore, our group has shown that microbubble cavitation is a crucial aspect for enhancing IN delivery, as the enhancement in the targeted region was only seen when FUS-mediated BBB opening was applied after IN delivery, and not conversely^18^. This “microbubble pumping effect” is believed to be the mechanism that enhances the delivery efficiency of IN molecules in targeted regions, however the exact mechanism has yet to be elucidated. Regardless of the exact underlying mechanism, by leveraging the increased delivery efficiency in a focal region, FUS + IN may allow for therapeutically relevant levels of drugs to be delivered to structures traditionally difficult for IN to reach.

As a continuation of our previous study in wild-type mice, we herein investigate the potential therapeutic efficacy of IN + FUS delivery of BDNF to an early-stage Parkinsonian mouse model. BDNF has been of interest for the treatment of neurodegenerative diseases, including PD, due to its importance to the survival and function of developed neurons^20^. Moreover, BDNF protein levels have been reported to be substantially decreased in the dopaminergic (DA) neurons in the substantia nigra (SN) of patients with PD^21^. A large body of literature supports BDNF as a therapeutic agent that can impede degeneration of DA neurons as well as stimulate dopamine production in areas affected by PD^22,23^. Furthermore, intrathecal infusion of the BDNF protein caused a reduction in nigrostriatal cell damage to the 1-methyl-4-phenyl-1,2,3,6-tetrahydropyridine (MPTP) induced Parkinsonism model in monkeys^24^. In combination with the fact that early stage PD patients have been shown to be highly responsive to DA stimulation^25^, localized delivery of BDNF to stimulate DA production in partially degenerated DA neurons may potentially offer a therapeutic strategy to halt or even reverse the progression of PD. Thus, the focus of this study was to evaluate the potential efficacy of IN + FUS delivery of BDNF for the treatment of neurodegenerative disorders such as PD. Specifically, we evaluated whether the expression of tyrosine hydroxylase (TH), the rate limiting enzyme in the biosynthesis of dopamine^26^, could be enhanced by IN + FUS delivery of BDNF in an early-stage PD mouse model. In this study, mice with bilateral degeneration of the nigrostriatal pathway received targeted unilateral treatments within one hemisphere. We hypothesize that any local changes in the treated nigrostriatal pathway would cause a biochemical and/or functional imbalance between hemispheres, which we elucidated through immunohistochemistry and behavioral testing.

## Results

### BDNF concentration increased in both the striatum and SN when FUS is properly targeted

**Figure 1:**
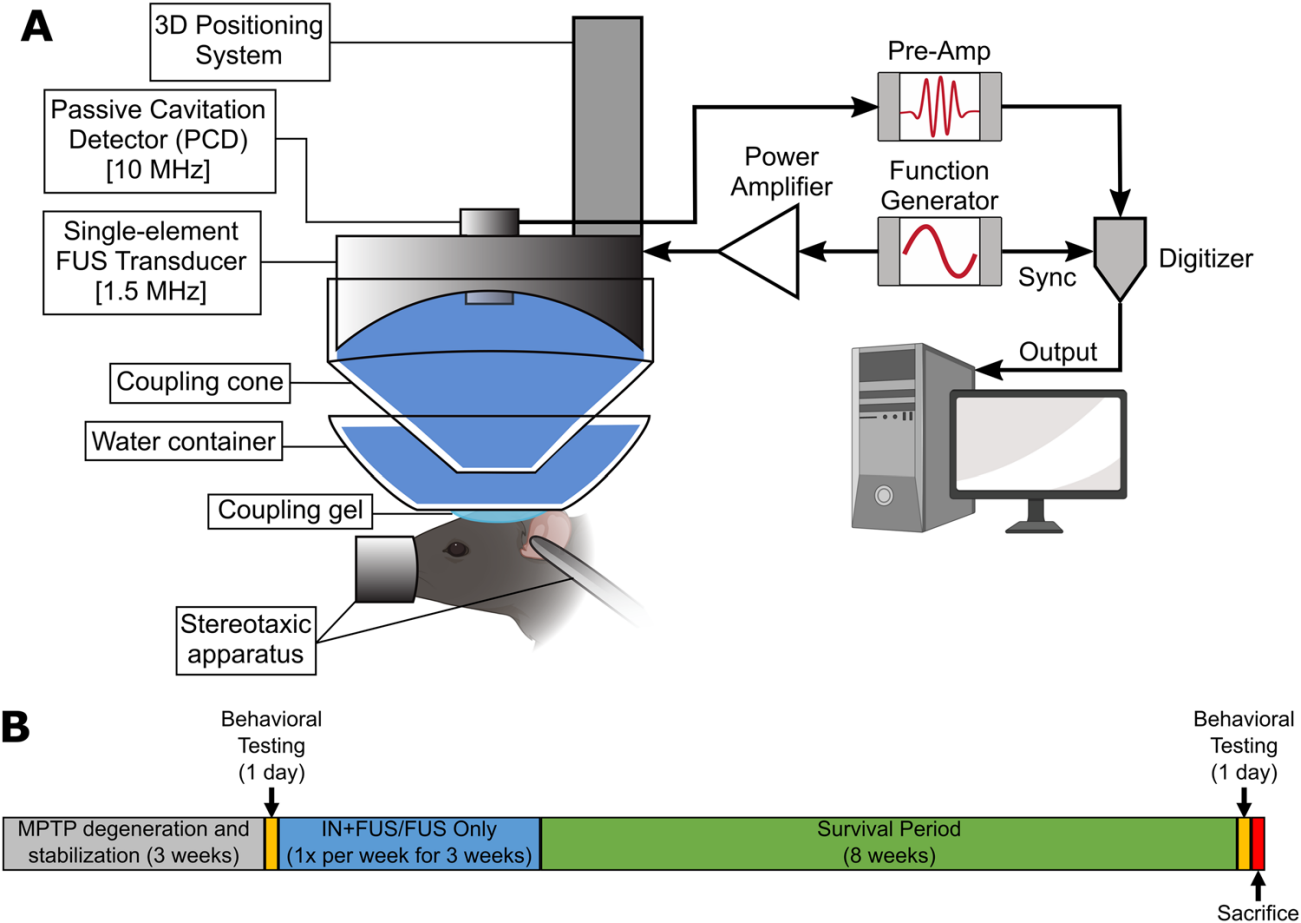
Experimental setup and study design. (**A**) Experimental setup for FUS-induced BBB opening with passive cavitation detection. (**B**) Timeline of the long-term study. Control MPTP mice receiving no FUS or IN BDNF were survived alongside the mice receiving treatment (FUS only/IN+FUS).

**Figure 2:**
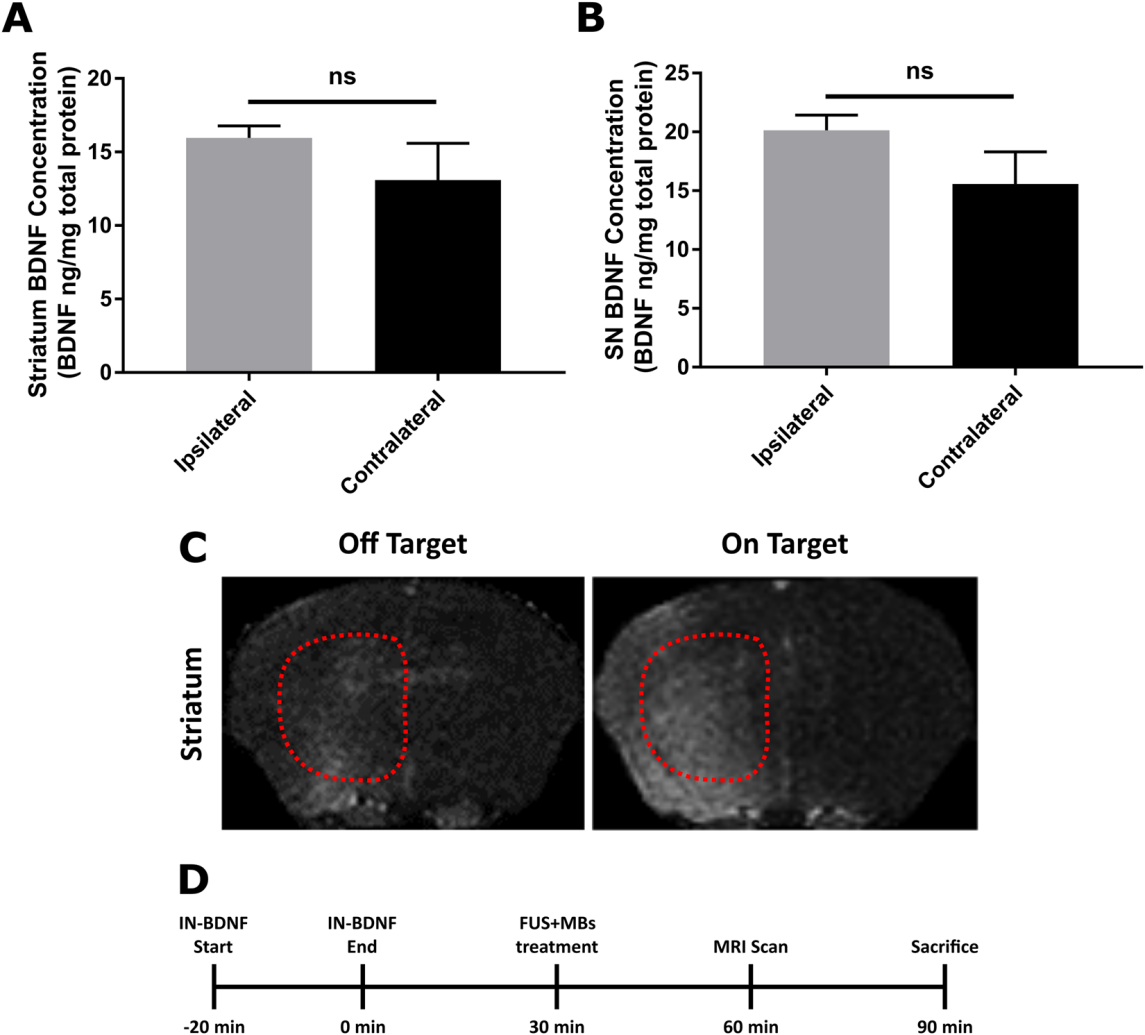
BDNF quantification confirming successful increase in BDNF in the targeted ipsilateral side. Total BDNF protein concentration per mg of total protein in the (**A**) striatum and (**B**) substantia nigra dissected from WT mice IN+FUS BDNF (n = 3). (**C**) Axial T1-weighted contrasted enhanced MRI showing examples of with poor and good targeting within the striatum (outlined by dotted red lines). Cases with poor targeting were excluded from the statistical analysis. (**D**) Experimental timeline for the short-term study to quantify BDNF delivery.

To determine successful localized enhancement of BDNF in the targeted regions, a short-term study was performed to quantify the amount of BDNF protein delivered in both the striatum and SN of wild-type mice after IN + FUS using the setup seen in Fig. 1A. Overall, BDNF quantification using ELISA (Fig. 2A,B) revealed a trend toward increased amounts of BDNF found in both the ipsilateral striatum and the SN when compared to the contralateral; however, these differences were found not statistically significant (p = 0.431 for the striatum, p = 0.2142 in the SN). When evaluating results from individual mice, those that did not have contrast enhancement in the targeted regions (off-target) did not see improvements over the contralateral side (Fig. 2C). However, mice with strong MR contrast enhancement in the accurately targeted region (on-target) saw approximately an increase of 35% in the striatum and a 38% in the SN increase in the amount of BDNF delivered when comparing the ipsilateral side to the contralateral side (n = 3). Mice for this short-term study followed the timeline shown in Fig. 2D.

### BBB opening and confirmation using Magnetic Resonance Imaging (MRI) and Passive Cavitation Detection (PCD)

**Figure 3:**
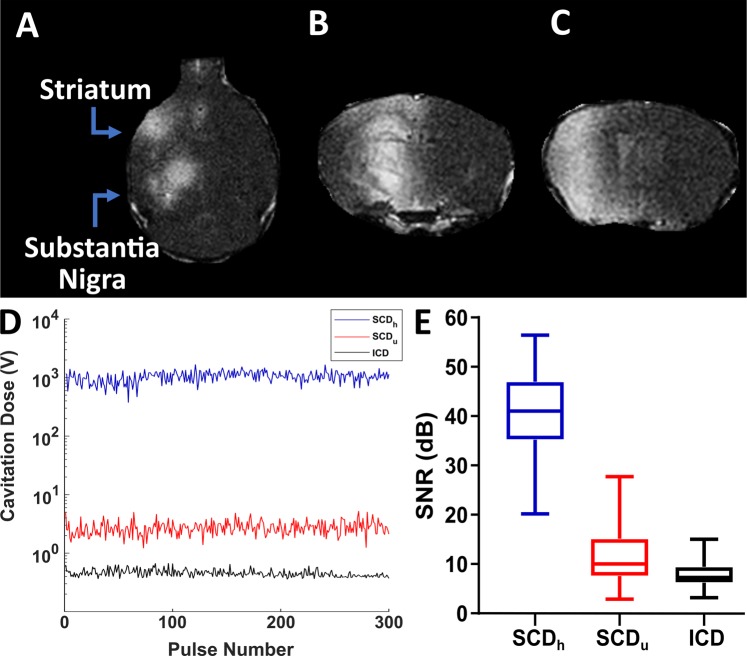
Effective BBB opening confirmation using T1-weighted contrast enhanced MRI and PCD measurements. (**A**) Representative axial MRI images showing enhancement in both targeted regions. Representative coronal MRI images showing enhancement in the (**B**) substantia nigra and in the (**C**) striatum. All MRI sequences were acquired approximately 40 min after injection of gadodiamide and qualitatively confirmed contrast enhancements in the targeted locations. (**D**) Cavitation dose for stable cavitation from harmonics (SCDh) and ultraharmonics (SCDu) as well as inertial cavitation dose (ICD) during one sonication (**E**) Measured stable and inertial cavitation for all mice receiving FUS induced BBB opening (n = 13). Cavitation dose with microbubbles was normalized to cavitation dose without microbubbles.

To confirm effective BBB opening and targeting, all mice that received FUS underwent T1-weighted contrast enhanced MRI to qualitatively confirm BBB opening and targeting accuracy. Over the three weeks of sonication, contrast enhancement was successfully seen in both the SN and striatum (Fig. 3A–C) in mice receiving FUS-induced BBB opening. Furthermore, PCD monitoring corroborated well with the BBB contrast enhancement, showing persistent stable cavitation activity during sonication and minimal inertial cavitation (Fig. 3D,E). Before the start of the 2^nd^ week of sonication, one mouse in the FUS only group died due to unknown circumstances. All mice in the other groups survived throughout the entire experimental timeline.

### IN + FUS BDNF significantly upregulates TH expression in the striatum

**Figure 4:**
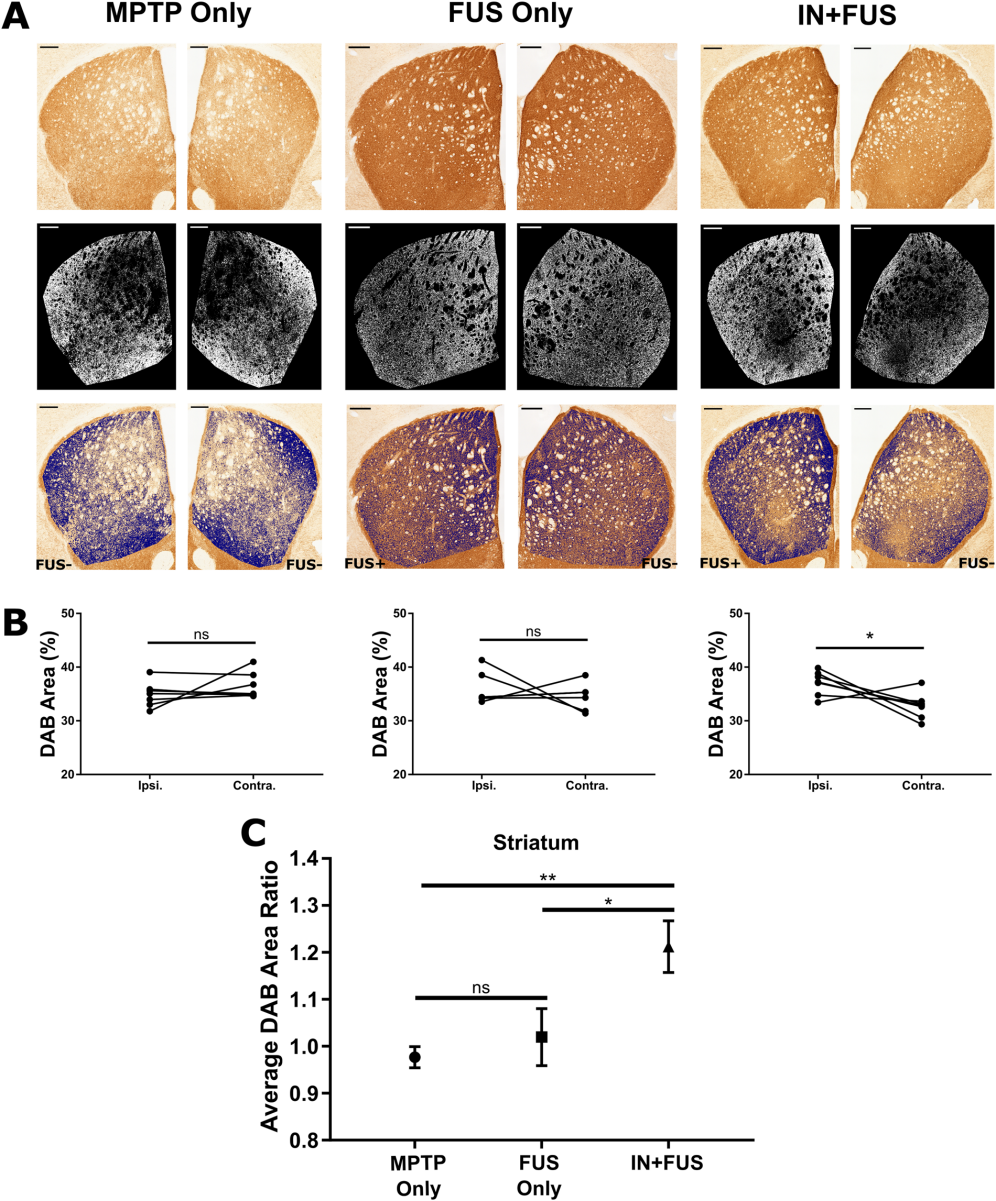
TH+ immunoreactivity in the striatum. (**A**) Representative images of the left and right striatum from each of the three groups showing raw image (top row), binary images of the extracted DAB staining from the raw image (middle row), and the overlay of the extracted DAB on the raw image (bottom row). The left hemisphere in the FUS only and IN+FUS groups received FUS induced BBB opening. Scale bar, 250 μm. (**B**) Average DAB area in the ipsilateral (left hemisphere) and contralateral (right hemisphere) striatum for each mouse for MPTP only (left, n = 7), FUS only (middle, n = 6), and IN+FUS (right, n = 7). Only the IN+FUS group showed a significant increase in DAB area when comparing ipsilateral and contralateral striatum (two-tailed paired Student’s t-test). (**C**) Average DAB area ratio from each group, taken by taking the normalizing the ipsilateral DAB area by the contralateral DAB area in each group. Average DAB area ratio for the IN+FUS group showed significant increases compared to both MPTP only and FUS only groups (One-way ANOVA with Holm-Sidak corrected post-hoc comparisons). All error bars represent standard error mean. * - p < 0.05, ** - p < 0.01, ns – not significant (p > 0.05)

Once FUS induced BBB opening was confirmed in all cases, the next step was to study the effect IN + FUS has on the DA neurons in the nigrostriatal pathway of MPTP mice. For the long-term study, these mice followed the experimental timeline shown in Fig. 1B. We first investigated the structural integrity of the nigrostriatal pathway terminal site at the striatum. In the sub-acute MPTP model, the remaining DA neurons that survived the MPTP toxin are severely impaired. The dysfunction of DA neurons can be seen in the downregulation of TH compared to normal mice. Therefore, we can use TH as a marker for structural integrity for these impaired DA neurons. Since unilateral treatment was applied, a difference in TH staining would be present in cases where an imbalance in local TH production was present between hemispheres. 3,3’-Diaminobenzidine (DAB) staining was used as a chromogen to visualize the TH stained (TH+) tissue. Staining of TH in the striatum was extracted (Fig. 4A) and the area of TH+ pixels in the ipsilateral and contralateral striatum for every mouse were compared (Fig. 4B). Within the MPTP only and the FUS only group, there was no statistical difference in average DAB area between ipsilateral and contralateral sides for each group (p = 0.2998 for MPTP only, p = 0.4997 for FUS only). The IN + FUS group, however, shows a significantly larger TH+ area in the ipsilateral side compared to the contralateral side (p = 0.0479). This indicates that the application of FUS-enhanced IN delivery of BDNF increase TH expression of the targeted striatal DA neurons compared to the untargeted striatal DA neurons, which acts as the baseline of IN delivery only. Furthermore, a one-way ANOVA for the three groups revealed a significant difference in DAB area ratio (p = 0.0056). The average DAB area ratio for each group shows that the MPTP and FUS only groups are close to 1.00, indicating similar TH expression on both sides of the brain, while the IN + FUS group has a ratio of 1.20 (Fig. 4C). Using Holm-Sidak’s post-hoc multiple comparisons test, this ~20% increase in the IN + FUS treated group is statistically significant when compared to both the MPTP only group (p = 0.0071) and the FUS only groups (p = 0.0243). Additionally, comparison of the DAB ratio between the MPTP and FUS only groups showed no statistically significant differences (p = 0.5417), indicating no adverse effects in the striatum due to FUS mediated BBB opening.

### IN + FUS BDNF moderately upregulates TH expression in the SN

**Figure 5:**
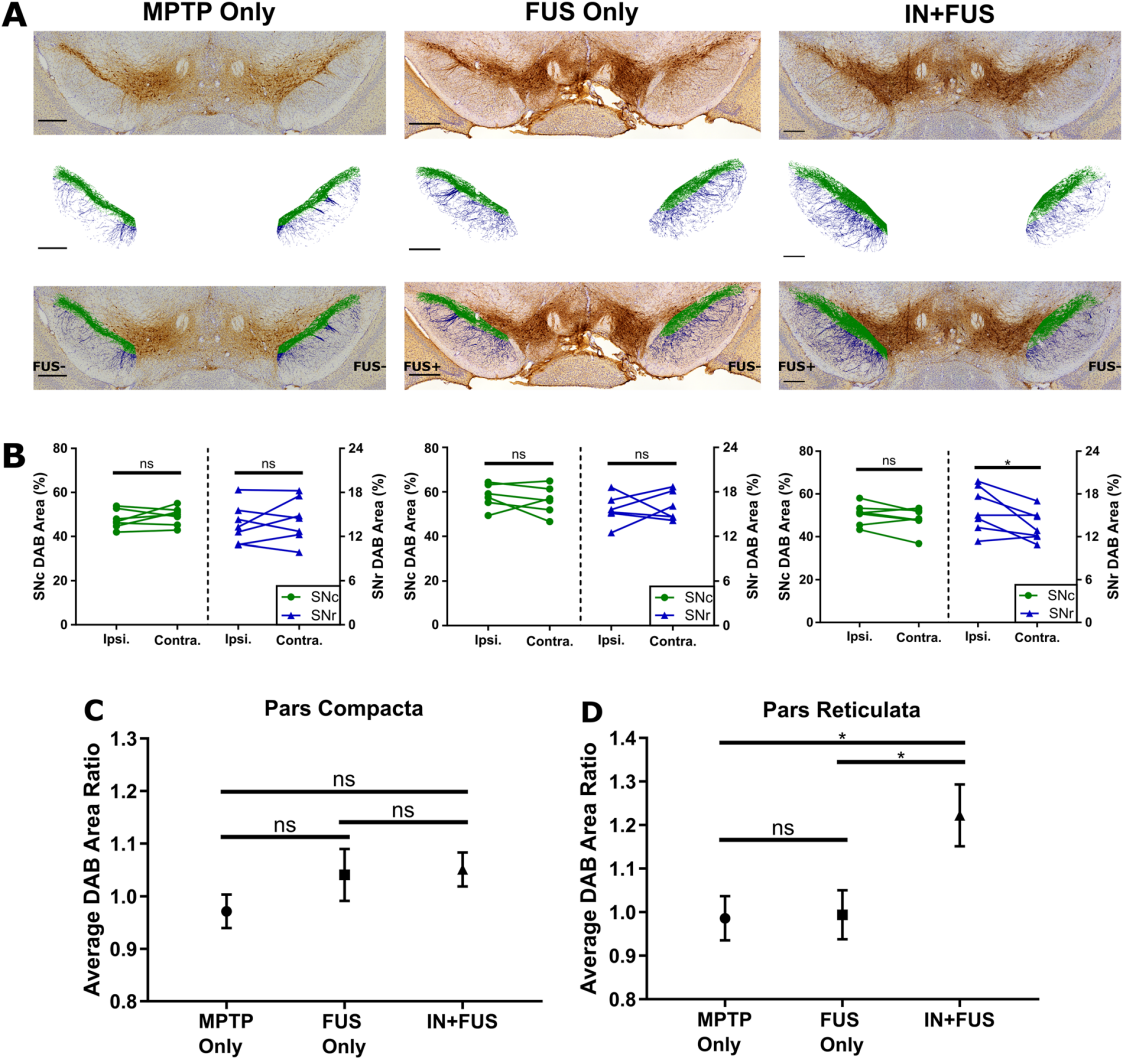
TH+ immunoreactivity in the SNc and SNr. (**A**) Representative images of the SN within in each experimental group showing raw images (top row), extracted DAB staining from the raw (middle), and overlay of the extracted DAB staining on the raw images (bottom). The extracted pixels taken from the SNc and SNr were colored green and blue, respectively. Scale bar, 250 μm. (**B**) Average DAB area for each mouse in the ipsilateral and contralateral in both SNc and SNr for MPTP only (left, n = 7), FUS only (middle, n = 6), and IN+FUS (right, n = 7). Only the SNr in the IN+FUS group showed significant differences between the ipsilateral and contralateral DAB area (two-tailed paired Student’s t-test). Average DAB area ratio for the (**C**) SNc and the (**D**) SNr, computed by normalizing the ipsilateral DAB area by the contralateral DAB area within each group. The IN+FUS group in the SNr showed significant increase in average DAB area ratio when compared to both the MPTP only and the FUS only group (One-way ANOVA with Holm-Sidak corrected post-hoc comparisons). Error bars represent standard error mean. * - p < 0.05, ns – not significant (p > 0.05).

After establishing improved TH immunoreactivity in the terminal field of the nigrostriatal pathway, we investigated the structural integrity of the cell bodies and dendrites in the SN region. Specifically, we determined the presence of TH+ immunoreactivity in both the substantia nigra pars compacta (SNc) and the substantia nigra pars reticulata (SNr). Applying the same intensity-based thresholding technique as previously used on the striatum, DAB stained pixels were extracted from both the SNc and SNr, and highlighted in green and blue, respectively (Fig. 5A). Focusing on the SNr, a comparison of the ipsilateral and contralateral DAB area for each mouse in each group (Fig. 5B, blue) revealed significant improvement in the ipsilateral hemisphere compared to the contralateral hemisphere for only the IN + FUS group (p = 0.0449). Among the three groups, a one-way ANOVA revealed a statistically significant difference in average DAB area ratio for the SNr (p = 0.0190). Moreover, multiple comparison post-hoc analyses showed that the average DAB area ratio in the SNr of 1.21 for IN + BDNF group was significantly greater than both the FUS only (p = 0.0351) and the MPTP only (p = 0.0343) groups (Fig. 5D). Within the SNc, no significant differences were found when comparing the ipsilateral and contralateral DAB area for each mouse in each group (Fig. 5B, green) as well as the average DAB area ratio (Fig. 5C). Changes in SNc were not expected given that the MPTP does not affect the cell body count ratio at this dose^27^. However, when comparing the average baseline DAB ratio for the MPTP only group, there is an increasing trend in the FUS only (ratio of 1.04) and IN + FUS groups (ratio of 1.06) within the SNc. Moreover, there were no significant differences, or decreasing trends, between MPTP only and FUS only groups in both the SNr and SNc, further confirming no harmful long-term effects on neuronal production of TH by the FUS induced BBB opening.

### IN + FUS BDNF improves functional behavior of the dopaminergic pathway

**Figure 6:**
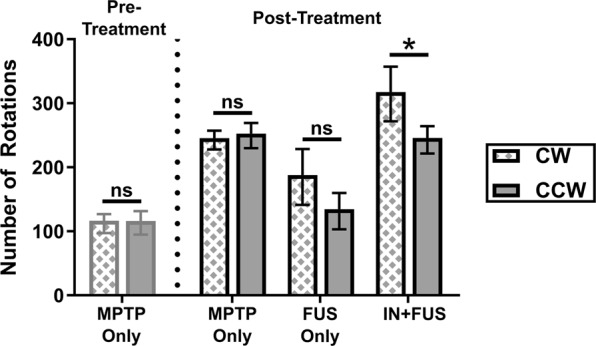
Absolute rotations during amphetamine elicited behavioral testing. Behavioral testing was performed after completion of MPTP lesioning, prior to the start of experimental procedures (pre-treatment) and 8 weeks after the last experimental procedure (post treatment). Pre-treatment MPTP only mice (n = 14) were separated into two cohorts that made up the post-treatment MPTP only (n = 7) and the IN+FUS groups (n = 7). FUS only group were treated taken from a separate cohort of MPTP mice (n = 6). Within each group, clockwise (CW) and counterclockwise (CCW) rotations were compared, with significant differences between CW and CCW rotation found only in the IN+FUS group (two-tailed paired Student’s t-test). Error bars represent standard error mean. * - p < 0.05, ns – not significant (p > 0.05).

With evidence of TH upregulation in both the striatum and SN of IN + FUS treated mice, we determined whether this upregulation translates to a functional b amelioration in these mice. By using amphetamine to stimulate the release and to block the reuptake of dopamine, we were able to elucidate whether there was a change in the nigrostriatal pathway functionality. With unilateral treatment, any changes to the local production of TH would introduce an imbalance in the nigrostriatal pathway that would in turn potentially induce a rotational bias in the treated rodents. Prior to any experimental treatment, mice injected with MPTP were tested. The pre-treatment MPTP mice have an average net rotation close to zero, with no significant differences between the number of CW and CCW rotations (Fig. 6). This reaffirms the fact that the sub-acute MPTP dose model causes symmetrical degeneration of the nigrostriatal pathway. Eight weeks after the final sonication/treatment, mice were subjected to amphetamine elicited rotational testing again. The MPTP only group continued to have a net rotation close to zero while both the FUS only and the IN + FUS group showed in increase in net rotations. One mouse in the MPTP only group showed a significant bias toward CW rotation, but was considered a significant outlier within the MPTP only group by the Grubb’s Outlier test (p < 0.05) and subsequently removed from analysis. When comparing the number of CW and CCW rotations within each group, statistically significant differences were only found in the IN + FUS group (p = 0.0403). This significant increase in net rotation (i.e greater number of CW rotations) in the IN + FUS group indicates an increase in DA release in the left hemisphere (treated hemisphere), indicating that the upregulation of TH in the treated hemisphere translate to functional improvement. Additionally, no decrease in net rotations in the FUS only group confirms that FUS mediated BBB opening alone did not cause any detrimental effects on the nigrostriatal pathway.

## Discussion

In this study, we investigated the potential therapeutic effects of FUS-enhanced IN delivery of BDNF in a Parkinsonian mouse model. Using an early-stage Parkinsonian mouse model, we delivered BDNF via the nasal passageway and further enhanced the delivery in targeted regions in the brain using FUS-induced BBB opening. The toxin-based MPTP model was chosen due to its reliable lesioning of the nigrostriatal pathway and the resemblance of neuropathological and clinical features of PD caused in humans, making the findings clinically relevant and potentially translatable^28^. Within the basal ganglia, some groups have proposed that the degeneration in PD follows a “dying-back” hypothesis, where the degeneration of nigral neurons begin in the distal axon and travels back to the cell body within the SN^29,30^. Additionally, clinical estimates of disease onset in PD patients occurred at ~30% loss of DA neurons in the substantia nigra^31,32^ and 50%-70% loss of DA neurons in the striatum^33^, further supporting the “dying-back” hypothesis. Moreover, these clinical findings support that the ~40% DA neuronal loss in the subacute MPTP dosing regimen^34,35^ recapitulate clinical findings in early-stage PD. As a result, using the subacute MPTP dosing regimen to emulate early-stage PD allows for the potential of neurorestoration of partially degenerated neurons that still have viable cell bodies but deteriorating axons and dendrites.

By employing the sub-acute MPTP dosing regimen, we have a nigrostriatal pathway that was not completely depleted, but instead composed of impaired DA neurons with subnormal expression of TH, the rate-limiting enzyme for DA synthesis. Subsequently, we first investigated if there were any changes in TH production within the nigrostriatal pathway on the terminal end and the cellbody/dendrites end. Any successful delivery of the neurotrophic factor BDNF to these dysfunctional DA neurons in the nigrostriatal pathway would potentially stimulate TH production. With treatment of IN + FUS, we found upregulation of TH+ cells in the nigrostriatal pathway compared to animals receiving MPTP only or FUS only. In the striatum, where the degeneration begins, we found a ~20% increase in the expression of TH in IN + FUS BDNF treated mice, a significant improvement compared to mice receiving MPTP only and MPTP mice receiving FUS induced BBB opening alone. This improvement indicates that BDNF was not only delivered to the brain through the IN route but also selectively released into the brain parenchyma within the regions targeted with FUS-induced BBB opening. This targeted release into the striatum allows BDNF direct access to the impaired DA neurons and for any therapeutic effects to take place. In the SN, where cell bodies of the DA neurons reside, we found similar improvement in the expression of TH. MPTP mice receiving IN + FUS saw a modest but not significant increase of 6% and a statistically significant increase of 21% in TH expression of the ipsilateral side compared to the contralateral side of the SNc and SNr, respectively. Though the increase in TH expression in the SN was not as prominent as that found in the striatum, it still indicates that BDNF was successfully delivered to the SN through IN + FUS. This decline in improvement may be due to the IN route itself, as the SN is furthest structure for any BDNF that has entered the brain via the olfactory bulb. Additionally, IN dosing may play an important role in the amount of BDNF reaching different regions, as a recent report found varying concentrations of [^125^I]-IgG reaching the striatum and midbrain of a rat after three different intranasal dosing regimens of [^125^I]-IgG^36^. Regardless, this modest improvement obtained in the SN indicates that once again IN + FUS was selectively released into the brain parenchyma and taken up by the DA neurons in the SN, increasing the local production of TH. From studying TH expression in the nigrostriatal pathway, we found that IN + FUS of BDNF was capable of increasing TH expression of DA neurons that have begun degeneration due to the MPTP toxin.

Next, we investigated if the upregulated TH expression in the treated nigrostriatal pathway translated to any behavioral changes. In our study, we employed amphetamine elicited rotational testing, the gold standard for testing behavioral changes in the 6-hydroxydopamine (6-OHDA) toxin-based PD mouse model. In the 6-OHDA model, toxins are intracranially injected to only one hemisphere, causing unilateral lesioning of the nigrostriatal pathway. Due to this asymmetry, when amphetamine is injected, an ipsilateral rotation towards the lesioned side is observed^37^. This amphetamine elicited rotational bias has been shown to correlate well with the integrity of the nigrostriatal pathway^38^. In the MPTP mouse model, amphetamine elicited rotational testing is not typically employed due to the toxin bilaterally lesioning the nigrostriatal pathway. However, in our study, treatment of IN + FUS BDNF was only applied to one hemisphere of MPTP mouse. If improvements in the nigrostriatal pathway of the treated DA were to occur neurons (i.e. increased production of DA), then an asymmetry of motor function would appear, resulting in a rotational bias when amphetamines are introduced. In our case, the rotational bias would be towards the untreated (contralateral) side, as it would be considered more lesioned compared to the side treated with IN + FUS BDNF. Furthermore, previous studies in literature have found that therapeutic effects of neurotrophic factors delivered to PD mouse models occurred on a time scale of weeks, ranging from 2 to 12 weeks^27,39,40^. As a result, the 8-week survival period chosen in the study was a suitable timepoint that was consistent with literature. From our study, rotational bias was only found in our IN + FUS treated group, with significantly greater rotations towards the contralateral side (CW rotation). This rotational bias indicates an imbalance in the DA pathway now exists in the IN + FUS treated mice, an asymmetry that did not exist prior to IN + FUS treatment. Both the FUS induced BBB opening group and the MPTP only group, did not see any statistically significant behavioral changes over the time course of the study. However, the FUS-induced BBB opening group did show a nonsignificant trend, due to insufficient power, toward greater CW rotations, suggesting a potential improvement in the DA pathway with FUS-induced BBB opening, as reported in prior studies^41^. This slight bias was also seen in previous workl^27^ and requires further investigation. Overall, these behavioral changes are indicative of the fact that the severity of lesioning on the ipsilateral side decreased due to the treatment of IN + FUS BDNF. Combined with the results from the first part of the study, we have shown that the upregulation of TH in the targeted basal ganglia translated to positive functional changes.

Due to the high costs associated with the amount of BDNF to each mouse, we were unable to devote resources to a group that received IN BDNF only. However, we can use the contralateral side of our IN + FUS treated group as a surrogate to the IN BDNF only group, as IN delivery alone of biologics has been shown to be able to be widely distributed throughout the brain^42,43^. Furthermore, we performed a meta-analysis in Table 1, comparing our results to others that have delivered BDNF intranasally only, and have found that overall IN + FUS has an increased delivery efficiency in targeted structures compared to other groups who have done IN only of BDNF. From these results, we can therefore infer that IN + FUS enhances the delivery efficiency of BDNF compared to IN alone in the subacute MPTP mouse model. Additionally, our group and others have shown that IN + FUS can enhance the delivery of various biologics, including BDNF, to targeted regions of the brain in wild-type mice^18,44^.

**Table 1:** Meta-analysis of previously reported *in vivo* BDNF measurements taken with and without IN delivery of BDNF.

Species	Total BDNF Delivered	*In vivo* BDNF Measurements	Time point	Method	Report
Mice	No BDNF	559.9 ng/g (hippocampus)	baseline	ELISA	^57^
Rats	No BDNF	636.1 ng/g (hippocampus)	baseline	ELISA	^57^
Mice	No BDNF	27.77 ng/g (striatum)	baseline	ELISA	^58^
—	—	29.48 ng/g (hippocampus)	baseline	ELISA	^58^
Rats	No BDNF	0.16 ng/g (whole brain)	baseline	ELISA	^59^
Rats	5 ug of rhBDNF	0.056 ng/L (hippocampus)	4 hours	Microdialysis	^60^
Rats	70 ug of [^125^I]-BDNF rhBDNF	1.7 ng/L (hippocampus)	1 hour	Gamma Counter	^61^
Mice	5 ug of [^125^I]-BDNF rhBDNF	0.05% inj/g (midbrain)	30 min	Gamma Counter	^62^
—	—	0.2% inj/g (striatum)	30 min	Gamm Counter	^62^
—	—	0.1% inj/g (whole brain)	5 min	Gamma Counter	^62^
Rats	40 ug of BDNF	4.33 ng/g (whole brain)	1 hour	ELISA	^59^
Mice	400 ug of rhBDNF	49.91 ng/mL || 29.05 ug/g || 7.2%/g (striatum)	1 hour	ELISA	This study
—	—	45.57 ng/mL || 35.07 ug/g || 8.7%/g (midbrain)	1 hour	ELISA	This study

BDNF measurements reported are of total BDNF in the specified structures.

Though the exact route of delivery of IN + FUS BDNF was not directly explored in the present study, results from this study and others further support the hypothesis that BDNF delivered intranasally is capable of being transported directly to the brain. Moreover, the unilateral improvement in TH expression in the targeted areas and the corresponding behavioral changes indicate that the applied FUS-mediated-BBB opening after IN BDNF contributed to improving the localized delivery of BDNF into the targeted regions. However, the exact route through which the BDNF reached the targeted location was not investigated in this study. The nasal mucosal is comprised of a rich bed of capillaries, allowing for the potential of BDNF to enter systemic circulation and enter the brain following FUS-mediated BBB opening^45^. Since we did not directly measure BDNF levels in the blood throughout the study, we cannot eliminate the possibility of systemic BDNF entering the brain after BBB opening. However, our previous studies have shown that in order for enhancement in the targeted regions, FUS-induced BBB opening must occur after IN delivery^18^. Therefore, most of the enhancement of BDNF delivery occurred during FUS-induced BBB opening, instead of after. Furthermore, though some BDNF is likely to have entered the systemic circulation through the nasal mucosal, it seems unlikely to have survived long enough in circulation before the BBB was opened due to the some literature reporting intrinsically short half-life of BDNF in circulation and the rapid clearance of it by the liver^46,47^. One interesting possibility that may occur after FUS-induced BBB opening is the diffusion of BDNF out of the brain through the opened BBB, back into systemic circulation. The ultrasound parameters used in this study for FUS-induced BBB opening were sufficient for IV administered BDNF to diffuse into the brain parenchyma^48^. Future studies are needed to determine whether, if any, FUS enhanced IN delivery of drugs leads into systemic drug circulation through the opened BBB, and whether it is possible to enhanced IN delivery using microbubble cavitation, without opening the BBB.

## Conclusion

From this study, we have shown that IN + FUS is a viable option for a non-invasive and localized technique for drug delivery. Additionally, the successful intranasal delivery of the BDNF through the barrier in an early-stage PD mouse model resulted in modest improvement in neuronal function that translated to quantifiable structural and functional motor changes. Through these findings, we have not only further supported that fact that neurotrophic factors such as BDNF may play a critical role in treatment of neurodegenerative disease, such as PD, but also further bolstered FUS-enhanced IN delivery as a potential non-invasive and effective drug delivery route to the CNS that may be clinically impactful.

## Materials and Methods

### Animal use and study design

All experimental procedures with animals were conducted in strict accordance with the Guide for the Care and Use of Laboratory Animals of the National Institutes of Health and were carried out under protocols approved by the Columbia University Institutional Animal Care and Use Committee. Male (6–8 week-old) C57BL/6 mice (Charles River, 25–30 gr) were housed under standard conditions with standard rodent chow and water *ad libitum*. The study consisted of two parts: (1) protein delivery confirmation in targeted regions of the brain and (2) protein delivery to a PD mouse model.

To confirm protein delivery in the brain, a total of three wild-type mice underwent IN + FUS delivery of BDNF. BDNF was administered to the mouse intranasally, followed by FUS-mediated BBB opening in the left SN and striatum. FUS targeting was confirmed using contrasted-enhanced magnetic resonance imaging (MRI). The animals were sacrificed 1.5 hr after the end of the IN treatment through decapitation and the brains were extracted immediately. From each hemisphere, the SN and striatum were dissected out and frozen immediately with dry ice and stored at −80 °C until protein quantification via enzyme-linked immunosorbent assay (ELISA). A summary of the timeline for the short-term quantification study can be seen in Fig. 2D.

For the second part of the study, a total of twenty-one mice underwent intraperitoneal (IP) injections of 1-methyl-4-phenyl-1,2,3,6-tetrahydropyridine (MPTP, 30 mg/kg free-base) for five consecutive days. This MPTP dosing regime has been previously published and is referred to as the sub-acute MPTP regimen, which causes bilateral apoptotic degeneration of the nigrostriatal DA pathway, resembling early-stage PD in patients^49^. Stabilization of the degeneration occurs 21 days after the last injection of MPTP, with an approximate 50% loss of striatal dopamine levels and up to 40% loss of DA neurons^34,35^. Post stabilization, the twenty-one MPTP mice were then randomly divided into three groups (n = 7/group): (1) MPTP control, (2) FUS-mediated BBB opening only (FUS), and (3) IN + FUS delivery of BDNF. The MPTP control group served as the baseline for quantifying the degeneration of the nigrostriatal pathway, while the FUS group served as the baseline for the effects of FUS-mediated BBB opening on MPTP mice. The FUS group received an intravenous injection of MBs prior to FUS induced BBB opening, while the IN + FUS group were treated the same as the wild-type mice used for protein quantification in the first part of the study. Following treatment, all mice underwent contrast enhanced MRI for FUS targeting confirmation. The timeline of our experimental protocol is depicted in Fig. 1B. Briefly, mice receiving FUS induced BBB opening were treated once a week for three consecutive weeks. Following the last week of treatment, all mice were kept alive for an additional eight weeks to allow for any neurorestorative effects to take place. The MPTP control group did not receive any FUS or BDNF and were maintained alongside the other two groups.

### Intranasal delivery of BDNF

Lyophilized recombinant human BDNF protein (ab206642, Abcam Inc., Cambridge, MA) was reconstitute with saline at a concentration of 16.67 mg/mL. While under isoflurane (1–2% at 2 mL/min of oxygen), mice were placed supine on an in-house-made holder which supports the head in a neutral horizontal position. Using a gel-loading pipette tip, a 3 $\mu$L liquid droplet of the BDNF solution was brought next to the nares of the mouse and allowed to be inhaled. This was repeated every 2 min to alternating nares for about 20 min. For each session, a total of 24 $\mu$L of the BDNF solution was delivered, equating to approximately 0.4 mg of BDNF protein delivered intranasally. Mice for the short-term study received a single intranasal dose of 0.4 mg of BDNF, while the MPTP mice that were treated for three weeks receive a total of approximately 1.2 mg of BDNF. This dosing concentration and scheme were chosen based on our prior studies delivery neurotrophic factors across the BBB. Using a similar dosing concentration, our previous study shows successful delivery of the neurotrophic factor neurturin via intravenous injections after FUS induced BBB to the same brain regions targeted here^50^. Moreover, our group recently has shown that the triple dosing regimen ameliorates the nigrostriatal pathway following FUS induced BBB opening and IV delivery of neurturin in mice receiving the same sub-acute MPTP dosing^27^. By utilizing prior successful methods, the chosen dosing concentration and scheme for this study were sufficient for the initial investigation of the efficacy of IN + FUS delivery of BDNF for treatment of PD.

### Focused Ultrasound (FUS) mediated BBB opening

Opening of the BBB was accomplished using FUS in combination with systemically injected size isolated lipid-shelled microbubbles. The experimental setup for FUS mediated BBB opening can be seen in Fig. 1A. In summary, a single-element, spherical FUS transducer (center frequency: 1.5 MHz, focal length 60 mm; diameter:60 mm, Imasonic, France) was driven by a function generator (33220A, Agilent, Palo Alto, CA) that was amplified using a 50-dB power amplifier (324LA, Electronic Navigation Industries, Rochester, NY). Concentrically aligned with the FUS transducer was another single element transducer (frequency: 10 MHz, focal length 60 mm, diameter 11.2 mm; Olympus NDT, Waltham, MA) that was used for passive cavitation detection (PCD) of microbubble cavitation during the FUS sonication. PCD monitoring was necessary for ensuring safe and effective BBB opening. PCD acquisition and processing was accomplished following established methodologies by our group^51^. Briefly, the PCD signals were transferred to a digitizer (Gage Applied Technologies Inc., Lachine, QC, Canada) that was then converted to the frequency domain using a fast Fourier transform. PCD analysis yielded three metrics characterizing microbubble activity: harmonic stable cavitation dose (SCD_h_), ultraharmonic stable cavitation dose (SCD_u_), and inertial cavitation dose (ICD). For each pulse, SCD_h_ and SCD_u_ were determined by calculating the root mean square (RMS) of the values surrounding the harmonic and ultraharmonic peaks, respectively, while ICD was calculated to be equal to the RMS of broadband signal amplitude between the harmonics and ultraharmonics. Only cavitation emissions emitted between 3 MHz and 9 MHz were used for PCD analysis.

For the groups that received FUS, BBB opening was induced only to the left hemisphere. FUS was first applied to the left substantia nigra (relative to lambdoid suture, 1.0 mm lateral, 1.3 mm anterior) followed by two non-overlapping locations in the left striatum (relative to lambdoid suture, 4.0 mm lateral, 3.0 mm anterior and 5.0 mm lateral and 2.0 mm anterior). The striatum received two sonications in order to completely cover the structure and to maximize the drug delivery. Each location was sonicated for 60 s, with a pulse repetition frequency (PRF) of 10 Hz, a pulse length (PL) of 10,000 pulses, and an estimated *in situ* peak negative acoustic pressure (PNP) of 0.45 MPa^52^. Prior to the first sonication in the SN, in-house size-isolated lipid shelled microbubbles (concentration: 8E8 bubbles/mL, diameter: 4–5), isolated using differential centrifugation^53^, were injected via the tail vein (dose: 1 *μL*/g of mouse). A short time (15–20 min) lapsed after the first sonication to allow circulating microbubble concentration to be cleared^54^, after which the same microbubble dosing regime was reinjected, and the striatum was sonicated in the two non-overlapping locations consecutively.

### Magnetic Resonance Imaging (MRI)

Mice that underwent FUS induced BBB opening received an MRI to confirm the targeting and opening of the BBB. A bolus injection of 0.2 ml of gadodiamide (Omniscan, GE Healthcare, Chicago, IL) was administered intraperitoneally immediately after the second sonication in the striatum. While under continued anesthesia (1–2% isoflurane), mice were placed in a birdcage coil (diameter 30 mm) and imaged using a 9.4 T MRI system (Bruker Biospin, Billerica, MA). Approximately 30–40 min after the gadodiamide injection, a T1-weighted 2D FLASH sequence was taken in the axial and coronal planes (TR/TE: 230/3.3 ms, flip angle: 70%, NEX: 6, resolution 100 *μ*m × 100 *μ*m, slice thickness: 400 *μ*m).

### Protein extraction for BDNF detection with ELISA

Frozen brains sections were weighed and 10 mL per 1 g of tissue lysis buffer was added to each. Lysis buffer consisted of T-PER solution (Thermo Fisher Scientific, Waltham, MA, USA) containing Halt Protease and Phosphatase Inhibitor Cocktail (Thermo Fisher Scientific, Waltham, MA, USA) and 10uL of inhibitors per 1 mL of lysis buffer. Samples were homogenized in an ice bath in intervals of 30 seconds, with 10 seconds cooling periods to prevent tissue heating. Homogenates were then centrifuged at 14,000 g for 30 min in 4 °C, supernatant was then recovered and stored at −80 °C until used for ELISA. Before the ELISA was carried out, the total protein concentration was determined by Bradford assay. The BDNF measurement was carried out using the Abcam Human BDNF ELISA kit following the manufacturer’s protocol (ab99978).

### Rotational based behavioral testing

To test for unilateral neurorestoration of the basal ganglia after treatment, mice underwent amphetamine-induced rotational behavioral testing^27,55^. Each mouse received an intraperitoneal injection of amphetamine (2.5 mg/kg, dissolved in saline). The mouse was then placed into a cylindrical, open-field chamber (diameter: 22.5 cm) and allowed to explore and acclimate to the open field for 10 min. A video camera placed directly above the open field chamber was used to record the mouse movements. After the initial acclimation period, the mouse movements were recorded for an additional 40 min, after which it was removed. Urine and feces were then counted and removed, and the chamber was cleaned with 70% ethanol and prepared for the next subject. A behavioral research tracking software (EthoVision XT 8.5, Noldus, Wageningen, Netherlands), was used to track and analyze the rotational behavior for each mouse. Total clockwise (CW) and counterclockwise (CCW) rotations were quantified through the software and the net rotations (total CW rotations – total CCW rotations) for each group are reported.

### Immunohistochemistry of dopaminergic neurons

All mice were sacrificed through transcardial perfusion with phosphate buffered saline followed by 4% paraformaldehyde. The brain was removed from the skull and left overnight to fix in 4% paraformaldehyde, after which it was switched to sucrose solution. Brains were frozen and sectioned coronally at 35 $\mu \,$m with a cryostat and all the sections containing the striatum and SN were collected. TH immunostaining was used to quantify the structural integrity of the DA pathway. Striatum and SN sections were incubated overnight in anti-TH antibody (ab657012, Millipore Sigma) solution at a dilution of 1:1000 and 1:2000, respectively. TH was visualized using the SuperPicture Polymer Detection Kit (878963, Thermo Fisher Scientific) and visualized with 3,3′-Diaminobenzidine (DAB) staining (D4293, Sigma-Aldrich). Additionally, SN sections were counter-stained with cresyl violet solution (1 gram of Cresyl Violet in 500 mL deionized water with 2.5 mL of 10% acetic acid). For each mouse, five to six coronal sections were performed across the striatum (AP + 0.98 mm to + 0.26 mm from Bregma) and SN (AP −2.92 mm to −3.80 mm from Bregma). Colored images taken on a Lecia Microscope (Leica DM6) at 10x magnification were used for TH staining quantification on MATLAB (2018b; Mathworks, Natick, MA, USA), using a custom algorithm discussed in the next section.

### Quantification of TH staining

Quantification of TH+ immunoreactivity in the SN and striatum was performed in MATLAB (2018b; Mathworks, Natick, MA, USA) following the algorithm outline below. In summary, the acquired colored images were white-balanced, color deconvolved to extract the dark brown DAB stain and converted to gray scale in order to isolate TH immunoreactive neurons in each region. Selection of regions of interests (ROI) in the SN and striatum were manually segmented based on a mouse atlas^56^. Furthermore, the SN was segmented into the pars compacta (SNc) and pars reticulata (SNr). The ipsilateral and contralateral pixels in each ROI were combined to determine the mean and standard deviation for each section. Each ROI was then thresholded using half a standard deviation below the mean for each respective ROI. As a result, for each section, there are different threshold values for the three ROI (striatum, SNc, SNr). This varying threshold between sections and ROIs was used to take into account the time sensitive nature of DAB color development. The TH+ area in each ROI was defined as the number of dark DAB pixels below the threshold normalized to the respective total ROI area. The ratio of the TH+ area of the ipsilateral side to the contralateral side was taken to assess any changes between the two hemispheres within each group. Average TH+ area for the ipsilateral and contralateral side for each mouse as well as the average TH+ area ratio for each experimental group is reported.

### Statistical analysis

All statistical analysis was conducted using the Graphpad Prism software (Version 7.03, GraphPad Software, La Jolla, CA, USA). All error bars are expressed in standard error mean (SEM). Intra-group analysis of rotational behavioral data and DAB area percentage were performed using a two-tailed paired Student’s t-test. Differences among groups for average DAB area ratio were analyzed using one-way analysis of variance (ANOVA) followed by post-hoc Holm-Sidak multiple comparisons correction. A significance level of 5% was used for all statistical analysis.
